# Development of Clinical Criteria for Functional Assessment to Predict Primary Nonfunction of High‐Risk Livers Using Normothermic Machine Perfusion

**DOI:** 10.1002/lt.25291

**Published:** 2018-10-25

**Authors:** Hynek Mergental, Barnaby T. F. Stephenson, Richard W. Laing, Amanda J. Kirkham, Desley A. H. Neil, Lorraine L. Wallace, Yuri L. Boteon, Jeannette Widmer, Ricky H. Bhogal, M. Thamara P. R. Perera, Amanda Smith, Gary M. Reynolds, Christina Yap, Stefan G. Hübscher, Darius F. Mirza, Simon C. Afford

**Affiliations:** ^1^ National Institute for Health Research Birmingham, Liver Biomedical Research Centre, College of Medical and Dental Sciences University of Birmingham; ^2^ Liver Unit Queen Elizabeth Hospital Birmingham, University Hospitals Birmingham National Health Service Foundation Trust; ^3^ Cancer Research UK Clinical Trials Unit, Institute of Cancer and Genomics Sciences University of Birmingham Birmingham United Kingdom

## Abstract

Increased use of high‐risk allografts is critical to meet the demand for liver transplantation. We aimed to identify criteria predicting viability of organs, currently declined for clinical transplantation, using functional assessment during normothermic machine perfusion (NMP). Twelve discarded human livers were subjected to NMP following static cold storage. Livers were perfused with a packed red cell–based fluid at 37°C for 6 hours. Multilevel statistical models for repeated measures were employed to investigate the trend of perfusate blood gas profiles and vascular flow characteristics over time and the effect of lactate‐clearing (LC) and non‐lactate‐clearing (non‐LC) ability of the livers. The relationship of lactate clearance capability with bile production and histological and molecular findings were also examined. After 2 hours of perfusion, median lactate concentrations were 3.0 and 14.6 mmol/L in the LC and non‐LC groups, respectively. LC livers produced more bile and maintained a stable perfusate pH and vascular flow >150 and 500 mL/minute through the hepatic artery and portal vein, respectively. Histology revealed discrepancies between subjectively discarded livers compared with objective findings. There were minimal morphological changes in the LC group, whereas non‐LC livers often showed hepatocellular injury and reduced glycogen deposition. Adenosine triphosphate levels in the LC group increased compared with the non‐LC livers. We propose composite viability criteria consisting of lactate clearance, pH maintenance, bile production, vascular flow patterns, and liver macroscopic appearance. These have been tested successfully in clinical transplantation. In conclusion, NMP allows an objective assessment of liver function that may reduce the risk and permit use of currently unused high‐risk livers.

**Abbreviations**
ALT: alanine transaminase
ATP: adenosine triphosphate
cDNA: complementary DNA
CI: confidence interval
CIT: cold ischemia time
CV: central vein
DBD: donation after brain death
DCD: donation after circulatory death
H & E: hematoxylin‐eosin
HBI: hypoxic brain injury
ICH: intracranial hemorrhage
ITU: prolonged intensive care unit stay
LC: lactate‐clearing
LFT: liver function test
miRNA: microRNA
NA: not applicable
NHS: National Health Service
NIHR: National Institute for Health Research
NMP: normothermic machine perfusion
non‐LC: non‐lactate‐clearing
PAS: periodic acid‐Schiff
PT: portal triad
qPCR: quantitative polymerase chain reaction
SD: standard deviation
SONOP: sonification solution
T0: sample taken shortly after commencing the perfusion
T6: sample taken after 6 hours of the perfusion
VITTAL: Viability testing and transplantation of marginal livers
WIT: warm ischemia time

The demand for donor organs in liver transplantation greatly exceeds supply, whereas the global incidence of end‐stage liver disease continues to rise, further increasing demand.^1^ In the United Kingdom during 2016‐2017, 19% of patients listed for liver transplantation were either removed from the waiting list (15%) or died (4%) within 1 year of listing.^2^ Despite the increasing utilization of grafts from donation after circulatory death (DCD) and high‐risk donation after brain death (DBD) donors, together known as extended criteria donors, wait‐list mortality has not decreased.^3^ Their use is associated with a higher incidence of early posttransplant complications such as primary nonfunction, early allograft dysfunction, and/or renal failure.^4^, ^5^, ^6^ Utilization of high‐risk organs remains low, with 159 out of 1041 livers procured in the United Kingdom during 2016‐2017 being discarded. Only 35% of all potential DCD livers were transplanted because of DCD donation failing to proceed, inconsistencies in interpreting donor history and laboratory results, macroscopic or histological assessment, surgeon experience, and the transplanting center’s expertise in marginal organ utilization.^7^, ^8^, ^9^, ^10^ These largely subjective factors impact upon the selection process and can compromise patient safety by resulting in the acceptance of high‐risk marginal grafts that fail to function, or conversely potentially usable organs being discarded due to a perceived risk of posttransplant complications.

Normothermic machine perfusion (NMP) of the liver is a novel technology developed to reduce ischemic damage and provide superior organ preservation compared with static cold storage. The purported advantages of NMP include the following:

1. Attenuation of ischemia/reperfusion injury.
2. Assessment of liver function prior to transplantation.
3. Improvement of transplant logistics.
4. The potential to deliver therapeutics to recondition currently unusable livers, enabling subsequent transplantation.^11^, ^12^, ^13^, ^14^

The aim of this study was to develop a standardized protocol for NMP, allowing functional assessment of donor livers rejected for transplantation, and to subsequently propose real‐time criteria that predict liver viability. Outcomes of functional assessment were then correlated with histopathological assessment, which is currently the gold standard to assess transplantability of extended criteria donor livers.

## Patients and Methods

### SOURCE OF DISCARDED HUMAN LIVERS

The study included 12 consecutively perfused livers offered to our team for research, regardless of cause, between May 2013 and June 2015. All organs were procured by the UK National Organ Retrieval Service, using standardized surgical protocols,^15^ with the primary intention of clinical transplantation and were subsequently declined by all UK centers. Ethical approval for the study was granted by the National Research Ethics Service Committee in London‐Surrey Borders (reference number 13/LO/1928). Consent to use donor tissues for research was obtained by specialist nurses in organ donation from the donor’s next of kin during consent for organ donation. All livers were preserved in University of Wisconsin preservation fluid and exposed to a variable period of static cold storage.

### NMP OF THE LIVER

The liver preparation for NMP was analogous to clinical transplantation. While bathed in slushed ice, any redundant tissues were removed. The portal vein was cleaned to its bifurcation and hepatic artery dissected to the gastroduodenal artery. Straight and curved 20‐Fr Medos cannulae were inserted into the celiac trunk and portal vein, respectively. Prior to commencing NMP, livers were flushed with 2 L of 10% dextrose solution at 37°C as per our unit’s transplant protocol. The liver was then placed into the machine’s reservoir, and the cannulae were primed with perfusion fluid and connected to the perfusion circuits. Where required, a wider artery, from the same donor and surplus to transplant requirements, was anastomosed to the existing hepatic artery to permit cannulation. NMP was performed using the Liver Assist device (Organ Assist, Groningen, the Netherlands), which provides dual perfusion of the hepatic arterial and portal venous systems, in a semiclosed circuit, using 2 rotatory pumps that produce pulsatile and nonpulsatile flows, respectively.

The initial pressure settings of 30 mm Hg for the artery and 8 mm Hg for the portal vein were increased to 50 and 10 mm Hg, respectively, within 30 minutes of commencing NMP. The pressure was set with the aim to maintain stable flows with adequate liver perfusion. However, in situations where the flows (in particular in the arterial circuit) were decreasing the perfusion pressures were raised in attempt to maintain adequate perfusion. The temperature was initially set to 25°C and increased incrementally to 37°C within 30 minutes. Oxygen was supplied via a Sechrist air/oxygen blender (S3500CP‐G, Inspiration Healthcare, Ltd., Leicester, UK). The fraction of inspired oxygen was set at 0.21 with 1 L of flow per minute across each oxygenator, in accordance with the manufacturer’s instructions. The perfusion fluid was based on 3 units of liver donor–specific blood group, Rhesus‐negative, packed red cells obtained from the UK National Health Service Blood and Transplant. The constitution of the perfusion fluid is detailed in Table 1.

**Table 1:** Perfusion Fluid Constitution

	Amount (Initial Bulk Fluid Administrated Into Reservoir)
Oxygen carrier	
Packed red blood cells	3 units
Drug	
Human albumin solution 5%	1000 mL
Heparin*	10,000 IU
Sodium bicarbonate 8.4%†	30 mL
Calcium gluconate 10%	10 mL
Vancomycin	500 mg
Gentamicin	60 mg
Continuous infusions	
Epoprostenol	2 μg/mL, commenced at 4 mL/hour and titrated as necessary
Intermittent drug administration	
Aminoplasmal 10%‡	50 mL bolus every 6 hours
Dextrose 10%	Infusion as necessary according to perfusate glucose concentration

*Bolus repeated every 3 hours.

†Bolus 10‐30 mL administrated if perfusate pH is <7.00 to maintain pH > 7.20.

‡Cernevit 2 mL and phytomenadione 1 mg (0.1 mL) added to Aminoplasmal 500 mL bottle.

### DATA AND SAMPLE COLLECTION PROTOCOL

**Figure 1:**
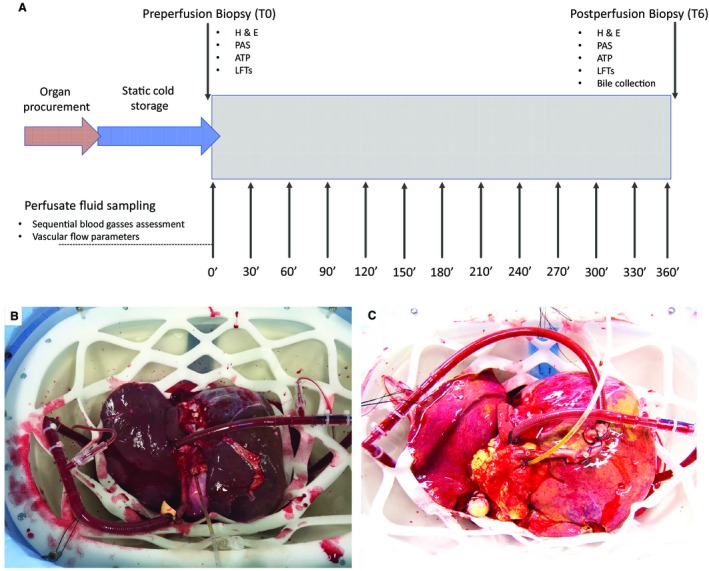
Study design and macroscopic appearance of a viable and nonviable liver. (A) The details of the study design and the perfusate fluid and biopsy sampling protocol. (B) A well‐perfused liver with optimal macroscopic appearance. The organ was rejected for transplantation due to the incidental discovery of a malignant melanoma. The liver began to function shortly after commencing the perfusion, and the vascular flows and blood gas profile patterns were used to help define criteria for liver graft viability (perfusion number 8). (C) A steatotic liver with suboptimal macroscopic appearance; this organ did not meet the viability criteria (perfusion number 2).

Flow rates, pressures, and resistances in the hepatic arterial and portal venous circuits were recorded every 30 minutes. Concurrently, 2 mL of perfusate from the arterial and venous circuits were collected for immediate blood gas analysis using the Cobas b 221 blood gas analyzer (Roche Diagnostics, Indianapolis, IN). If produced, bile was collected cumulatively and weighed at the end of the procedure. Liver biopsies were taken immediately prior to starting NMP, at 3 hours, and either after 6 hours or at the end of NMP, whichever was earlier. The tissue sample was divided and fixed in formalin as well as snap‐frozen in liquid nitrogen. The summary of the sampling protocol is shown in Fig. 1A.

### ASSESSMENT OF PHYSIOLOGY

The perfusate from the arterial and venous outflow was analyzed to measure partial pressures of O_2_ and CO_2_, pH, base excess, bicarbonate, O_2 _saturation, hemoglobin, hematocrit, sodium, potassium, chloride, calcium, glucose, and lactate concentrations. A perfusate pH < 7.00 was corrected using 20‐mL boluses of 8.4% sodium bicarbonate. Oxygen consumption per gram of liver tissue was calculated based on oxygen delivery and oxygen extraction from the arterial and hepatic venous elements of the circuit, respectively. The oxygen extraction ratio was calculated as the ratio of oxygen consumption to oxygen delivery.

### HISTOPATHOLOGICAL ASSESSMENT

After paraffin embedding and processing, liver biopsies were stained with hematoxylin‐eosin (H & E) and periodic acid‐Schiff (PAS). Biopsies were assessed for preexisting acute or chronic liver injury, large and small‐droplet macrovesicular steatosis, coagulative necrosis, intrahepatic bile duct injury (apoptosis, vacuolation, and lifting of epithelium from the basement membrane), hepatocyte plate injury (hepatocyte loss of cohesion, detachment of hepatocyte plates from the sinusoidal lining), and glycogen depletion, which were recorded as percentages of cells affected.^16^ Histological assessment was conducted by independent experienced liver transplant pathologists who were blinded to the designated viability.

For ultrastructural examination by transmission electron microscopy, 2‐mm biopsy pieces were fixed in 2.5% glutaraldehyde and processed to a resin block, and photomicrographs taken at ×13,000 magnification of mitochondria within random hepatocytes and examined for signs of injury.^17^

### ASSESSMENT OF ADENOSINE TRIPHOSPHATE

Measurements of adenosine triphosphate (ATP) were performed from snap‐frozen tissue by immediate homogenization in a sonification solution (SONOP) buffer (0.372 g of ethylene diamine tetraacetic acid in 130 mL of H_2_O and NaOH [pH 10.9] + 370 mL of 96% ethanol) using the GentleMacs system. Protein concentration was determined using a Pierce bicinchoninic acid (BCA) Protein Assay kit (Thermo Scientific Inc., Rockford, IL). An ATP Bioluminescent Assay kit (FLAA, Sigma‐Aldrich Inc., St. Louis, MO) was used to determine concentrations from a calibration curve on the same plate, corrected for amount of protein and expressed as nmol/g protein.

### ASSESSMENT OF LIVER CELLULAR DAMAGE BY microRNA ANALYSIS

The extent of the liver damage was estimated by microRNA (miRNA) 122 quantitative polymerase chain reaction (qPCR) analysis. RNA was isolated using Qiagen RNeasy kits (Eqicon, Vedbaek, Denmark) with the inclusion of Exiqon synthetic Spike‐in templates as controls.^18^ On column deoxyribonuclease digestion eliminated genomic DNA. RNA samples were assessed on a TapeStation (Agilent Technologies, Inc., Santa Clara, CA) using 10‐ng RNA per complementary DNA (cDNA) synthesis reaction with Exiqon cDNA synthesis reagents (miRCURY LNA Universal miRNA PCR kit) on a Labcycler (SensoQuest, Gottingen, Germany). Real‐time PCR was performed on a Roche LC480 using the miRCURY LNA Universal real‐time miRNA PCR kit following reagent and protocol guidelines. C_t_ values were generated via the Absolute Quantitation and second derivative methods, and relative quantities were calculated.

### STATISTICAL METHODS

A total of 27 perfusion parameters were recorded over a 6‐hour period at approximately 30‐minute intervals (details shown in Supporting Table 1). These were plotted against time, giving each liver its own observable trajectory, enabling trends to be visualized. Because of the small sample size, mean, standard deviation (SD), and range have been presented at initiation of NMP and then after 2, 4, and 6 hours of perfusion (Table 2).

**Table 2:** Liver Functional Assessment Parameters

	Non‐LC				LC			
Time (hours)	0	2	4	6	0	2	4	6
Lactate, mmol/L	13.7 (4.1) [7.2‐20.0]	14.6 (5.7) [4.4‐20.0]	13.7 (4.5) [9.2‐20.0]	14.6 (5.7) [6.9‐20.0]	10.5 (3.3) [5.5‐13.9]	3.0 (1.7) [0.6‐5.5]	2.1 (1.1) [0.7‐4.0]	2.1 (1.1) [0.7‐3.1]
Glucose, mmol/L	49.3 (9.7) [37.2‐64.1]	50.5 (9.7) [39.5‐64.5]	40.3 (12.4) [26.2‐60.3]	34.1 (15.2) [15.1‐56.2]	36.4 (18.3) [9.3‐56.6]	41.3 (12.9) [23.3‐59.3]	34.1 (14.8) [14.2‐56.7]	29.6 (20.2) [8.0‐52.4]
pH	7.3 (0.5) [6.8‐8.0]	7.3 (0.3) [6.8‐7.8]	7.4 (0.4) [6.9‐7.8]	7.4 (0.2) [7.2‐7.8]	7.2 (0.2) [6.9‐7.5]	7.3 (0.1) [7.2‐7.4]	7.4 (0.1) [7.3‐7.6]	7.4 (0.1) [7.3‐7.6]
Arterial flow, mL/minute	213.8 (238.5) [11.0‐593.0]	316.3 (224.4) [103.0‐631.0]	412.6 (269.7) [98.0‐810.0]	495.2 (330.8) [136.0‐835.0]	155.0 (96.2) [58.0‐313.0]	524.2 (118.8) [426.0‐727.0]	575.2 (43.1) [527.0‐638.0]	621.2 (52.1) [550.0‐682.0]
Arterial flow rate (mL/minute/g)	0.1 (0.1) [0.01‐0.5]	0.2 (0.1) [0.1‐0.3]	0.2 (0.1) [0.1‐0.4]	0.3 (0.2) [0.1‐0.5]	0.1 (0.05) [0.03‐0.2]	0.3 (0.1) [0.2‐0.4]	0.3 (0.1) [0.2‐0.4]	0.3 (0.1) [0.3‐0.4]
Portal flow, mL/minute	613.3 (177.7) [470.0‐910.0]	962.5 (261.5) [690.0‐1250.0]	1120.0 (70.7) [1030.0‐1210.0]	1158.0 (125.2) [970.0‐1320.0]	458.3 (166.8) [210.0‐630.0]	1176.0 (192.3) [1000.0‐1430.0]	1330.0 (225.1) [1100.0‐1650.0]	1418.0 (320.3) [1070.0‐1920.0]
Portal flow rate (mL/minute/g)	0.3 (0.1) [0.2‐0.4]	0.5 (0.1) [0.4‐0.7]	0.6 (0.1) [0.4‐0.7]	0.6 (0.1) [0.4‐0.7]	0.2 (0.1) [0.1‐0.4]	0.6 (0.2) [0.4‐0.9]	0.7 (0.1) [0.5‐0.9]	0.7 (0.3) [0.5‐0.9]
Hematocrit, %	29.0 (1.4) [27.8‐31.1]	23.3 (5.4) [14.8‐29.5]	16.0 (4.4) [11.3‐20.8]	16.6 (6.0) [12.1‐23.4]	26.2 (3.0) [20.8‐29.7]	22.6 (1.3) [21.3‐24.0]	21.3 (2.0) [18.9‐23.5]	19.8 (2.3) [17.7‐23.0]
Oxygen consumption, mL/minute	24.2 (1.4) [23.2‐25.2]	34.1 (7.8) [25.1‐39.4]	23.8 (13.7) [7.3‐39.6]	46.8 (11.1) [31.0‐57.0]	15.0 (13.3) [1.4‐37.0]	34.2 (17.4) [15.8‐58.5]	32.3 (15.7) [18.5‐83.9]	54.2 (28.1) [18.5‐83.9]
Oxygen consumption, mass	0.013 (0.002) [0.011‐0.014]	0.017 (0.005) [0.013‐0.023]	0.013 (0.006) [0.004‐0.017]	0.027 (0.010) [0.018‐0.041]	0.008 (0.008) [0.001‐0.021]	0.018 (0.009) [0.008‐0.029]	0.016 (0.008) [0.005‐0.024]	0.027 (0.011) [0.011‐0.036]
Oxygen extraction ratio	0.2 (0.1) [0.2‐0.3]	0.2 (0.04) [0.2‐0.3]	0.3 (0.3) [0.2‐0.8]	0.3 (0.2) [0.2‐0.6]	0.2 (0.1) [0.02‐0.3]	0.2 (0.1) [0.1‐0.3]	0.2 (0.1) [0.1‐0.2]	0.3 (0.1) [0.1‐0.4]

NOTE:

Data are given as mean (SD) [range].

The effect of lactate‐clearing (LC) and non‐lactate‐clearing (non‐LC) liver status on the change in liver function parameters (lactate and glucose metabolism, pH, arterial and portal flow rates, hematocrit, oxygen extraction ratio, and oxygen consumption) were explored through multilevel linear models for repeated measures. Random intercept and slope effects were assigned at the liver level. Where linear relationships were not observed, data were transformed as appropriate. Explanatory variables bicarbonate, carbon dioxide, and base excess were adjusted for in the pH model; hepatic artery pressure and hepatic artery resistance were adjusted for in the hepatic artery flow rate model; and portal vein pressure and portal vein resistance were adjusted for in the portal vein flow rate model. An indicator variable based on LC trajectories and its interaction with time were included in each model and included if found to be significant. For these exploratory analyses, because the sample size is small, any potential interactions between lactate clearance and time with *P* value <0.2 would be presented. Models were estimated using the method of maximum likelihood estimation and selected using likelihood ratio tests.

Missing data were recorded as follows: lactate 7.7%; glucose, arterial, and portal flow rates 8.3%; pH 11.5%; hematocrit 18.6%; and oxygen extraction ratio and oxygen consumption 20.5%. The used multilevel models approach used is tolerant of missing data under a missing at random assumption. Multilevel modeling was performed using Stata, version 14.2 (StataCorp LLC, College Station, TX).

The bile production, ATP, and miRNA levels were compared with the Mann‐Whitney U test, with the statistical level of significance set at *P *< 0.05, using GraphPad Prism (GraphPad Software, La Jolla, CA) software.

## Results

### DONOR DEMOGRAPHICS, CHRONOLOGY, AND REASONS FOR DISCARDING LIVERS

Eight livers included in the study were from DCD donors. The median donor age was 56 (range, 30‐76) years, and the median body mass index was 30 (range, 23‐47) kg/m^2^. The median cold ischemia time (CIT) was 483 (range, 380‐797) minutes. Of these livers, 3 were discarded because of steatosis, 2 for extrahepatic primary donor malignancy, 2 for excessive CIT, and 2 for excessive donor warm ischemia time (WIT). The detailed characteristics of the included livers are provided in Table 3.

**Table 3:** Donor Demographics and Chronology

	Non‐LC^*^						LC^*^					
Liver number	1	2	3	4	5	6	1^†^	2	3	4	5	6^†^
Donor age, years	55	55	76	60	46	71	30	69	55	57	70	50
Donor sex	Female	Male	Female	Female	Male	Male	Male	Male	Male	Male	Female	Female
BMI, kg/m^2^	47	33	28	36	23	30	25	31	24	25	34	45
Blood group	B+	A+	O+	A+	O+	O‐	A+	O+	O+	A+	O+	O+
Cause of death	Meningitis	ICH	ICH	HBI	ICH	HBI	HBI	HBI	Meningitis	ICH	HBI	HBI
Donor type	DBD	DCD	DCD	DCD	DBD	DCD	DCD	DBD	DBD	DCD	DCD	DCD
Agonal period, minutes	NA	14	8	17	NA	31	100	NA	NA	14	16	29
Primary WIT, minutes	NA	12	17	15	NA	12	12	NA	NA	14	18	11
Liver weight, grams	2420	2130	1775	1712	1961	2310	1997	2400	2300	1752	1650	1943
Steatosis assessment^††^	Moderate	Moderate	Nil	Moderate	Mild	Moderate	Nil	Mild	Nil	Mild	Mild	Nil
CIT, minutes	792	797	554	491	380	467	445	496	454	532	583	408
Donor risk index	1.85	2.64	3.23	2.77	1.41	3.22	1.77	1.78	1.61	2.36	3.05	2.39
Reason for discard	Steatosis	Steatosis	CIT^§^	Steatosis	ITU	Perfusion^||^	WIT^¶^	Fibrosis	Cancer	Cancer	CIT§	WIT¶

NOTE:

Agonal period in DCD procurement was defined as the period between withdrawal of treatment to circulatory arrest. Primary WIT in DCD procurement designs time from circulatory arrest to in situ organs perfusion.

^*^ The livers are grouped according the lactate metabolism (viability criteria) rather than the chronological order of the perfusion.

^†^ Designates livers that were transplanted.

^††^ Subjective assessment by the retrieval and/or transplant surgeon.

^§^ Prolonged CIT.

^||^ Poor quality liver graft perfusion.

^¶^ Extensive WIT and CIT.

### LIVER FUNCTIONAL ASSESSMENT

**Figure 2:**
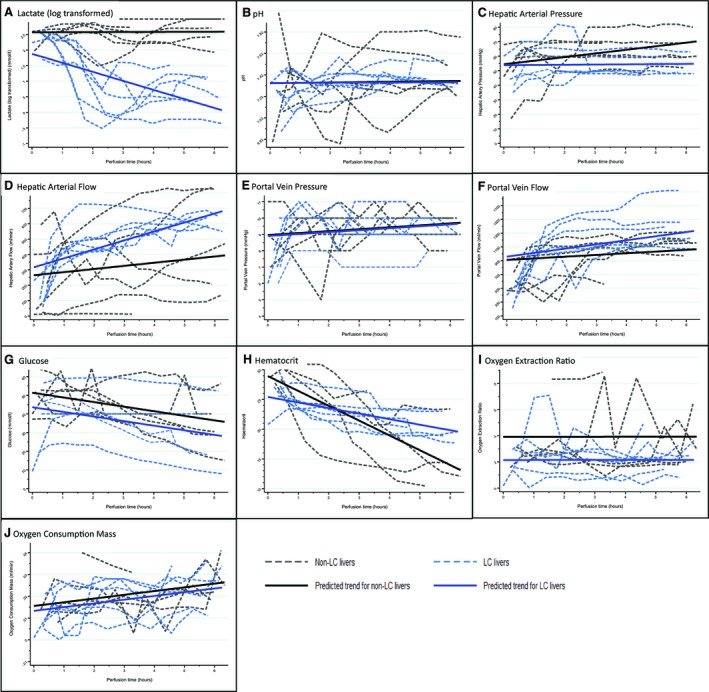
Multilevel random intercept and slope model findings. (A‐H) Graphs illustating each liver response trajectory over time (dashed lines) with corresponding average trajectory predicted from the multilevel model (solid lines) for LC and non‐LC livers. (A) Log‐transformed lactate levels (mmol/L): a significant difference in trend over time (*P *< 0.001) was observed, with LC livers being lower in comparison to non‐LC livers. (B) The pH: on average, LC livers appear to have a gentler increasing trend compared with non‐LC livers (*P* = 0.10), after adjustment for bicarbonate, carbon dioxide, and excess base. (C) Hepatic arterial pressure (mm Hg): the trends were different with a much steeper increasing trend in the non‐LC livers (*P* = 0.08), after adjusting for pressure and resistance. (D) Hepatic artery flow (mL/minute): there appears to be a difference in trends between LC and non‐LC groups (*P* = 0.13) after adjusting for hepatic arterial pressure, hepatic arterial resistance, and their interactions. (E) Portal vein pressure: an increasing trend over time (*P* = 0.07) was observed, but there was no difference between LC and non‐LC livers. (F) Portal vein flow (mL/minute): portal flow increased over time (*P* = 0.13), with LC livers having a slightly higher increment in flow (*P* = 0.12), after adjusting for pressure and resistance. (G) Glucose levels (mmol/L): glucose levels decreased significantly over time (*P* = 0.006) and LC livers appear to have lower levels compared with non‐LC livers. (H) Hematocrit: hematocrit demonstrated a significant reduction over time (*P *< 0.001) with LC livers showing a gentler decreasing trend (*P* = 0.01). (I) Oxygen extraction ratio: the levels were found not to change significantly over time, but on average, LC livers were 0.2 units lower than non‐LC livers (*P* = 0.07). (J) Oxygen consumption (mL/minute/g): a significant increase in oxygen consumption mass over time was observed (*P *< 0.001); however, there appears to be no difference between LC and non‐LC livers.

Initial graphical data explorations were performed with the aim of observing any trends over time. Individual livers’ response data were recorded for lactate, glucose, arterial and portal flows, pH, oxygen extraction ratio, oxygen consumption, and hematocrit (Fig. 2; Table 2).

The results for lactate measurements showed 2 distinct groups; 1 had a sharp fall in lactate levels that subsequently stabilized at lower levels, designated as the LC group, whereas the other showed fluctuations and rises in the lactate level over time, known as the non‐LC group. No other response variable measured showed a similar performance demarcation, although the LC livers did appear to show similarities of behaviors when plotted.

### MULTILEVEL RANDOM INTERCEPT AND SLOPE MODELS

Results from multilevel modeling found that lactate levels demonstrated a significant difference in trend over time (*P *< 0.001), with LC livers being lower in comparison to non‐LC livers.

After adjusting for bicarbonate (*P *< 0.001), carbon dioxide (*P *< 0.001), and excess base (*P *< 0.001), pH levels increased over time (*P* = 0.003), although LC livers appear to have a gentler increasing trend compared with non‐LC livers (*P* = 0.10). There was a difference in the trend of hepatic arterial pressure over time (*P* = 0.08) with a much steeper increasing trend in the non‐LC livers compared with the LC livers (*P* = 0.08). Changes in arterial flow, after adjusting for arterial resistance (*P* = 0.007) and arterial pressure (*P* = 0.14) and their subsequent interaction (*P* = 0.01), showed a slightly higher increasing trend in LC livers over time (*P* = 0.13). Portal vein pressure showed an increasing trend over time (*P* = 0.07). However, there was no significant difference between LC and non‐LC livers (*P* = 0.90). Portal vein flow increased over time (*P* = 0.13), with LC livers having a higher increment in flow rate (*P* = 0.12) after adjusting for portal vein pressure (*P *< 0.001), portal vein resistance (*P* = 0.25), and their interactions (*P* = 0.001). Glucose levels decreased significantly over time (*P* = 0.006), with LC livers being 7.8 mmol/L lower on average compared with non‐LC livers (*P* = 0.15). Hematocrit demonstrated a significant reduction over time (*P *< 0.001) with LC livers showing a gentler decreasing trend (*P* = 0.01). The oxygen extraction ratio was found not to change significantly over time, but on average for LC livers, it was 0.2 lower than non‐LC livers (*P* = 0.07). A significant increase in oxygen consumption over time was observed (*P *< 0.001). However, there appears to be no difference between LC and non‐LC livers (*P* = 0.85). The multilevel model parameters are provided in Table 4.

**Table 4:** Multilevel Random Effects Model Parameters Examining Liver Response Variables During Perfusion

Response Variables	Explanatory Variables	Estimate (95% CI)	*P* Value
Lactate (log) (mmol/L)	Time (hours)	0.003 (–0.1 to 0.1)	0.96
	LC indicator	–0.7 (–1.2 to –0.2)	0.005
	Interaction: LC indicator × time	–0.3 (–0.4 to –0.2)	<0.001
pH	Time (hours)	0.003 (0.001 to 0.006)	0.003
	LC indicator	0.002 (–0.02 to 0.02)	0.85
	Interaction: LC indicator × time	–0.002 (–0.005 to 0.0004)	0.10
	CHCO3	–0.05 (–0.06 to –0.05)	<0.001
	pCO2	–0.006 (–0.008 to –0.05)	<0.001
	Base excess	0.06 (0.06 to 0.06)	<0.001
Hepatic artery pressure (mm Hg)	Time (hours)	2.5 (0.7 to 4.3)	0.008
	LC indicator	–0.6 (–14.0 to 12.8)	0.93
	Interaction: LC indicator × time	–2.3 (–4.9 to 0.2)	0.08
Hepatic artery flow (mL/minute)	Time (hours)	20.4 (–15.2 to 55.9)	0.26
	LC indicator	51.7 (–91.6 to 195.0)	0.48
	Interaction: LC indicator × time	38.0 (–10.6 to 86.6)	0.13
	Hepatic artery pressure	–2.3 (–5.4 to 0.8)	0.14
	Hepatic artery resistance	–224.9 (–387.1 to –62.7)	0.007
	Interaction: pressure × resistance	3.3 (0.8 to 5.7)	0.01
Portal vein pressure (mm Hg)	Time (hours)	0.1 (–0.01 to 0.2)	0.07
	LC indicator	–0.06 (–1.0 to 0.9)	0.90
Portal vein flow (mL/minute)	Time (hours)	24.5 (–6.8 to 55.8)	0.13
	LC indicator	48.6 (–36.9 to 134.1)	0.27
	Interaction: LC indicator × time	34.9 (–8.6 to 78.5)	0.12
	Portal vein pressure	163.4 (108.4 to 218.4)	<0.001
	Portal vein resistance	21,183.4 (–14,933.6 to 57,300.4)	0.25
	Interaction: pressure × resistance	–6972.7 (–11,140.1 to –2805.3)	0.001
Glucose (mmol/L)	Time (hours)	–2.5 (–4.2 to –0.7)	0.006
	LC indicator	–7.8 (–18.3 to 2.8)	0.15
Hematocrit (%)	Time (hours)	–2.5 (–3.4 to –1.6)	<0.001
	LC indicator	–3.5 (–7.4 to 0.4)	0.08
	Interaction: LC indicator × time	1.6 (0.3 to 2.8)	0.01
Oxygen extraction ratio	Time (hours)	0.0002 (–0.02 to 0.02)	0.98
	LC indicator	–0.2 (–0.4 to 0.01)	0.07
Oxygen consumption (mL/minute)	Time (hours)	3.8 (1.7 to 5.8)	<0.001
	LC indicator	–1.4 (–16.5 to 13.6)	0.85

### BILE PRODUCTION

There were significant differences in cumulative bile production between LC and non‐LC groups. There was more sustained bile production in the LC group, although this only occurred in 4 livers. In the non‐LC group, only 1 liver produced bile at the end of the NMP (2.6 g at 6 hours). After 6 hours, the median bile production for LC and non‐LC groups was 6.5 versus 0.0 g (*P* = 0.03), respectively.

### HISTOLOGICAL FINDINGS

There was a significant discrepancy between the subjective assessment of liver quality performed by the organ retrieval or transplant surgeon and the subsequent histological findings. Microscopic evaluation confirmed only mild large‐droplet macrovesicular steatosis in livers declined for steatosis. Histology did not reveal any fibrosis in the liver declined for this presumed diagnosis.

**Figure 3:**
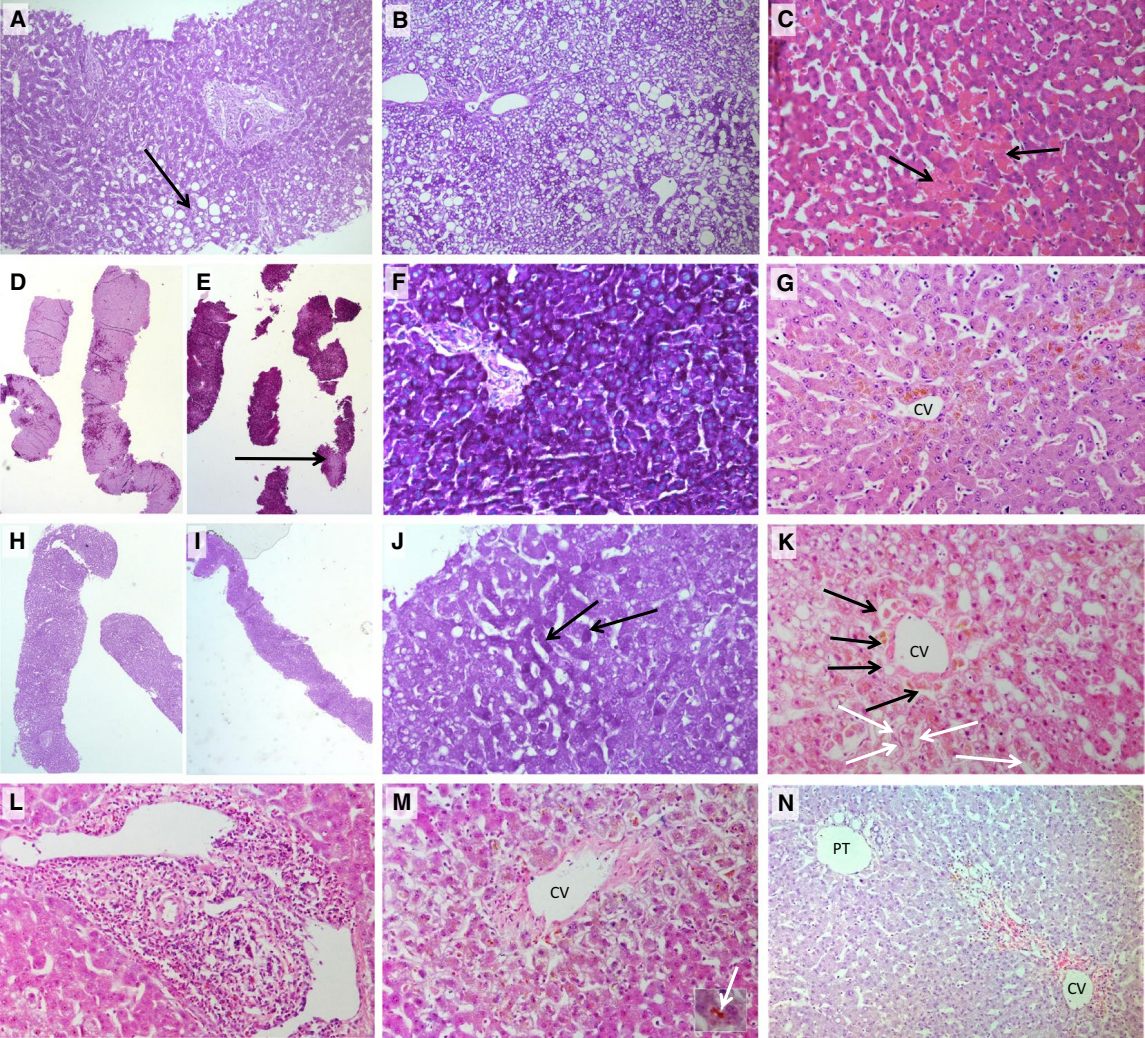
Histological findings. (A) A PAS‐stained section of a non‐LC liver, 4 of which had the most severe large‐droplet macrovesicular steatosis (arrow), the type of fat considered in evaluating suitability for transplantation. This was mild involving up to 15% of hepatocytes. The liver was turned down on macroscopic assessment of steatosis (original objective ×10). (B) A PAS‐stained section of liver 1 before NMP with extensive small‐droplet microvesicular steatosis, where hepatocyte cytoplasm contains often numerous small droplets of fat that do not displace the hepatocyte nuclei. Several large fat droplets are also present. This liver was turned down due to the macroscopic appearance of steatosis; large‐droplet steatosis was mild involving only 5% of hepatocytes in the whole biopsy. It is likely that the small‐droplet steatosis was also seen macroscopically. This is not traditionally considered in assessing a liver for transplantation and indicates the requirement of a liver biopsy to accurately assess the type and amount of both types of fat droplets (original objective ×10). (C) A H & E–stained section of LC liver 1 at 6 hours after NMP, showing a small area of coagulative necrosis where the cells become hypereosinophilic (arrows). This was seen to an equal extent in both viable and nonviable livers before and after NMP and was very mild in this series of livers. (D‐F) PAS stain from LC liver 1. (H‐J) Non‐LC liver 4. (D and H) Both livers demonstrated marked glycogen depletion pre‐NMP; although after NMP (E and F), the viable liver has restored its glycogen stores. (I and J) The nonviable liver remains significantly glycogen depleted. Bright magenta staining of the cytoplasm indicates glycogen, and pale pink staining indicates no glycogen (arrow; E). (J) The few darker staining hepatocytes containing some glycogen are indicated (D, E, H, I, original objective ×2; F, J, original objective ×20). (G) A LC liver 3 after 6 hours of NMP, revealing normal hepatocyte plate morphology and attachment of hepatocyte plates to the CV. (K) Non‐LC liver number 3 showing loss of cohesion of hepatocytes from each other and from the sinusoidal lining (arrows) and the CV 6 hours after NMP. (L and M) H & E–stained sections of non‐LC liver 5, which was turned down for transplantation based on its macroscopic appearance. This liver had (L) portal hepatitis and (M) severe zone 3 cholestasis (inset—high power of bile plug, arrow; original objective ×20 for both). (N) H & E–stained section of LC liver 2 discarded because macroscopically thought to have fibrosis. There is no fibrosis present. There is a normal portal triad (PT) showing no fibrous expansion. The abnormality present is centered around the CV consisting of confluent areas of hepatocyte loss in which there is variable hemorrhage/congestion (red color of red blood cells seen) and pigment laden macrophages (original objective ×10).

None of the livers displayed significant large‐droplet steatosis and at most showed only a mild degree (maximum of 15%; Fig. 3A). Small‐droplet macrovesicular steatosis was greater in the non‐LC livers (Table 5; Fig. 3B). Ischemic‐type coagulative necrosis was minimal across both groups (Fig. 3C). Lost cohesion of hepatocytes, predominantly in zone 3, was observed in the non‐LC group (Fig. 3K) with all post‐NMP livers showing variable amounts of hepatocyte detachment (LC 1.5% [0%‐10%] versus non‐LC 15% [1%‐40%]).

**Table 5:** Histological Features on Liver Biopsies

	Non‐LC						LC					
Perfusion number	1	2	3	4	5	6	1^*^	2	3	4	5	6^*^, ^§^
Large‐droplet steatosis, %^†^	5	5	<5	15	0	0	0	0	0	0	0	<1
Small‐droplet steatosis, %^††^	90	30	<5	40	0	0	5	10	0	0	0	10
Glycogen depletion, %^§^ (pre/post NMP)	30/90	75/90	99/80	90/80	—	5/10	80/15	—	5/5	85/75	40/30	95/10
Detached hepatocytes, %^||^ (pre‐NMP/post‐NMP)	0/1	4/30	0/40	20/15	—	0/5	0/1	—/0	0/0	10/10	0/5	1/2
Bile duct injury^¶^ (pre‐NMP/post‐NMP)	0/2	0/2	0/1	0/0	—	0/1	0/1	—/0	0/0	0/1	0/0	0/0
Coagulative necrosis, %^#^ (pre‐NMP/post‐NMP)	0/1	0/0	0/0	0/5	—	0/10	0/0	—/0	0/0	0/2	0/10	0/5
Other findings	Microthrombi	Mild portal hepatitis		Patchy congestion	Hepatitis with severe cholestasis	Mild portal edema with eosinophils		l‐2 week‐old lytic zone 3 necrosis				
Time of 2nd biopsy (hours)	6	3.2	6	6	6	6	4.5	6	6		6	5

NOTE:

Values designated with “—” are missing.

^*^ Designates livers that were transplanted.

^†^ Large‐droplet macrovesicular steatosis is defined as a single large fat droplet within the hepatocyte cytoplasm displacing the nucleus; values are % of hepatocytes containing fat.

^††^ Small‐droplet macrovesicular steatosis is defined as fat droplets, usually multiple, within the cytoplasm of the hepatocyte that do not displace the nucleus; values are % of hepatocytes containing fat.

^§^ Glycogen depletion is graded as the % of hepatocytes that do not contain glycogen.

^||^ Detached hepatocytes is the % of hepatocytes that have lost cohesion from each other and from the sinusoidal lining.

^¶^ Bile duct injury is defined as apoptotic debris within the wall or lumen or loss of cohesion between the epithelium and basement membrane; it is graded as 0 (nil), 1 (minimal), and 2 (present).

^#^ Necrosis is depicted as the percent of total hepatocytes in the biopsy that shows classical ischemic‐type coagulative necrosis.

There was no difference in amount of glycogen depletion before NMP between the groups (Fig. 3D,H; LC 80% depletion, 5%‐95% versus nonviable 75% depletion, 5%‐99%). At the end of the perfusion, the LC group displayed increased PAS staining (Fig. 3E versus Fig. 3I; LC 22.5% glycogen depletion, 5%‐80% versus non‐LC 80% depletion, 10%‐90%), indicating that viable livers were able to uptake glucose and store this as glycogen (Fig. 3F) or maintain glycogen stores if initially high.

The intrahepatic bile ducts displayed greater injury, in particular apoptosis of biliary epithelial cells, in the non‐LC group (median of 1 versus 0) compared with the LC group. Detailed histological findings are shown in Table 5.

**Figure 4:**
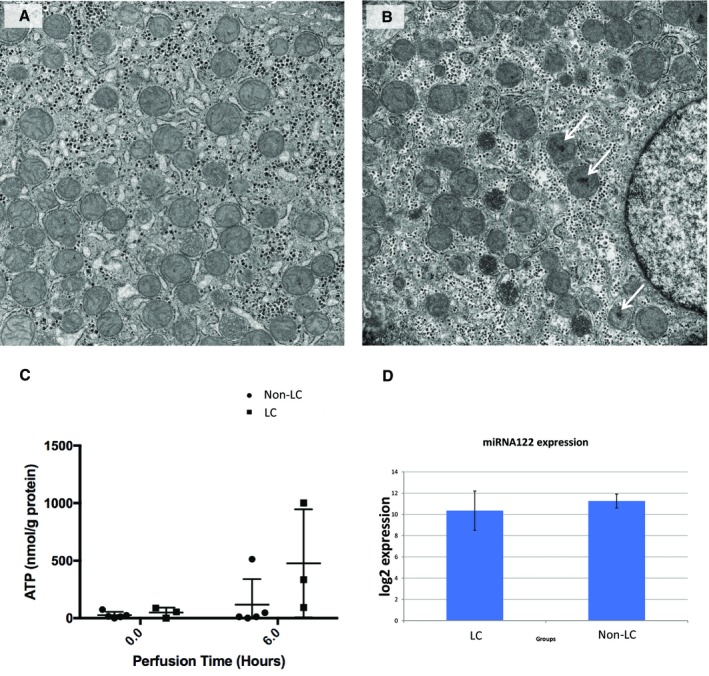
Transmission electron micrographs and ATP and miRNA analyses. (A) shows a LC, viable liver number 4, and (B) a non‐LC liver number 6. Both microphotographs were taken from postperfusion (T6) biopsy samples. In the nonviable liver, flocculent densities can be seen within several of the mitochondria (white arrows), which indicate irreversible cell injury. Christae are still apparent within other mitochondria and within the viable liver (A) in which no flocculent densities were observed. The mitochondria of both livers are not swollen (original magnification ×13,000). (C) Preperfusion and postperfusion ATP levels, showing increase in the LC livers contrasting with minimal change observed in non‐LC livers. (D) MiRNA assays to assess the extent of cellular damage. This analysis did not reveal any difference between LC and non‐LC groups.

The ultrastructural assessment by transmission electron micrograph demonstrated the mitochondria were not swollen in either liver group. However, flocculent densities, a sign of irreversible cell injury, were present in many mitochondria in the non‐LC livers but were not present in the LC livers (Fig. 4).

### ATP FINDINGS

The ATP analysis was performed from 8 livers, showing nonsignificant differences between median preperfusion levels (54.6 versus 15.8 nmol/g; *P* = 0.42), followed by a trend for increasement in the LC livers at 6 hours, contrasting with reduced ATP levels in the non‐LC group (334.6 versus 11.9 nmol/g; *P* = 0.18). Details are shown in Table 6.

**Table 6:** Liver Perfusion Parameters and Proposed Viability Criteria

	Non‐LC						LC					
Liver number	1	2	3	4	5	6	1^*^	2	3	4	5	6^*^
Perfusion time (minutes)	541	192	501	1102	738	394	393	277	378	403	388	316
Lactate T0 (mmol/L)	>20.0	13.4	13.0	13.3	7.2	15.2	7.6	9.4	12.9	13.9	13.9	5.5
Lactate T2 (mmol/L)	19.2	16.4	20.0	12.5	4.4	15.1	1.2	4.6	0.6	5.5	3.2	3.0
Trough lactate (mmol/L)	12.8	13.4	13.0	8.8	4.4	6.9	0.7	2.1	0.6	1.2	0.8	1.4
Bile production T6 (grams)	0.0	0.0	0.0	0.0	2.6	0.0	23.0	6.1	10.4	0.0	6.9	0
ATP T0 (nmol/g protein)^†^	15.8	—	12.1	0.0	24.6	74.7	—	—	54.6	88.1	0.0	—
ATP T6 (nmol/g protein)^†^	46.6	—	0.6	11.5	11.9	512.8	—	—	334.6	1001.9	93.5	—
ALT (IU/L)† T0	—	—	4055	—	—	2888	—	—	574	—	2603	3673
ALT (IU/L)† peak value			—			5017			1498	10,772	3803	6851
Major criteria: Trough lactate level of <2.5 mmol/L Presence of bile production												
Minor criteria: Perfusate pH of >7.30 Stable arterial flow of more than 150 mL/minute and portal flow more than 500 mL/minute Homogeneous liver perfusion with soft consistency of the parenchyma												

NOTE:

A viable liver graft has to meet ≥1 major and ≥2 of the minor criteria. All parameters are assessed 120 minutes after commencing the perfusion. To ensure recipient safety and to minimize risks of presence of a preexisting liver disease or irreparable liver damage, only organs meeting the following criteria were considered for the pilot clinical transplant series: maximum donor age of 70 years, CITs of <16 hours for livers from donors after brain death, or <10 hours from DCD, donor WIT (systolic blood pressure <50 mm Hg to aortic perfusion) in DCD organs <60 minutes, absence of hepatitis B, hepatitis C, or human immunodeficiency virus infection, and healthy macroscopic appearance without signs of fibrosis or cirrhosis (Mergental et al.^12^).

a ^*^ Designates livers that were transplanted.

^†^ Values designated with “—” are missing.

### ASSESSMENT OF LIVER CELLULAR DAMAGE BY miRNA ANALYSIS

For the purpose of the assay, Sp6 was used as the interplate calibrator and Sp4 as the internal amplification control. Outliers with C_t_ values >37 were excluded. The samples were normalized to the reference gene miRNA‐23b, converted to relative quantities and a log scale. Preprocessed normalized data did not reveal any difference between LC and non‐LC livers (*U* value of 39; *P* = 0.25)

### VIABILITY ASSESSMENT CRITERIA

Two livers were declined for unexpected malignancy confirmed in other organs after the retrieval. These 2 livers had a favorable macroscopic appearance (Fig. 1B) and donor characteristics, and during NMP, they demonstrated properties expected of livers after transplant, enabling us to propose perfusion parameters associated with functioning livers.

The ability of livers to clear lactate appeared to be a substantial marker to divide the livers into 2 groups. Bile production was closely related to lactate clearance. However, its negative predictive value was low.

In defining clinically usable viability criteria to assess the function of high‐risk and/or discarded livers, our main objective was to ensure transplant recipient safety. We designed a composite viability measure consisting of lactate clearance and/or bile production (major criteria), in combination with additional minor criteria of stable arterial and portal flows, perfusate pH, and favorable macroscopic assessment by the transplant surgeon (Table 6; Fig. 2).

## Discussion

NMP has been developed to overcome shortcomings and organ damage occurring during static cold storage. Preserving the liver in near‐physiological conditions at normothermia, with oxygen and nutrients, allows for ex vivo functional assessment. Our key objective when commencing the NMP program was to develop a protocol to evaluate liver function and define criteria characteristic of a viable liver with a view to preventing primary nonfunction while using high‐risk extended criteria organs. This research, performed on discarded donor livers that had been exposed to a variable period of static cold storage, assumed that during NMP potentially transplantable livers would behave similarly to an allograft following its implantation. Two livers in the study had, barring incidental donor malignancy, otherwise favorable donor characteristics and macroscopic appearances, with NMP commencing after a short duration of CIT. Provided favorable perfusion characteristics had been observed, if postprocurement biopsies from the suspicious donor tissues had not shown malignancy, these organs could have been transplanted. The demonstrated perfusion characteristics and metabolic activity in these 2 livers were similarly observed in 4 other livers. The most striking functional indicator in these 6 livers was their ability to metabolize lactate to near physiological levels within 2 hours of NMP, a quality not seen in the other 6 high‐risk livers. This LC group was expected to consist of viable, transplantable livers. The remaining 6 non‐LC livers were deemed nonviable. A more detailed analysis of the liver perfusion characteristics showed livers that metabolized lactate were more likely to maintain a physiological pH without intervention, establish physiological flow rates in both the hepatic artery and portal vein, and have a less declining hematocrit. We also added evidence of bile production because this is generally accepted as a favorable indicator of graft function, although its absence is not proof of nonfunction. Using a composite of these parameters was aimed at maximizing patient safety.

The present study reveals unique data and novel observations. These are the first criteria to be successfully tested in clinical practice and subsequently adopted within a clinical study of viability testing and transplantation of discarded human livers.^12^ The criteria are easy to measure and consist of familiar parameters. Lactate concentration is an important indicator of graft function in the peritransplantation period, and as such, its inclusion facilitated clinical adoption of the protocol.^19^ This is the first report that includes marginal organs that were so severely damaged that we were unable to maintain the perfusion for 6 hours, which has enabled us to assess the full spectrum of liver function. The proposed criteria appear to correlate closely with the current gold standard assessment of liver transplantability: histopathological assessment. We have demonstrated the quite marked variability in the assessment of steatosis by the retrieving or transplanting surgeon and the histology of the liver. In this era of progressive organ shortage, such inconsistency may contribute to the waste of potentially usable livers, further highlighting the urgent need to develop objective assessment methods to improve the relatively low utilization of high‐risk organs.

The Groningen group was the first to demonstrate the feasibility of NMP on 4 discarded human livers. The livers were subjected to 6 hours of NMP following a median CIT of 6 hours 55 minutes, with all organs showing recovery of function and being deemed viable.^20^ The inferior outcomes of some livers from our series may be explained in part by the CIT being on average 2 hours longer. A subsequent study from the Groningen group reported an NMP series on 12 discarded livers, proposing 6 hours of cumulative bile production >20 g as a marker of good liver function.^21^ We were unable to define a cutoff volume because some viable organs in our series did not produce bile. We concur with the observation reported by Sutton et al. of significantly lower lactate levels in the livers with a high bile output.

The Cambridge group advocated assessment based on perfusate transaminases and bile pH.^14^ The authors observed a significant correlation between the alanine transaminase (ALT) in the perfusate measured after 2 hours’ perfusion and the peak ALT posttransplant levels within the first week.^14^ They also hypothesized that the liver’s capacity to produce an alkaline bile (pH > 7.4) might be a good marker of cholangiocyte function, possibly identifying a selection of organs with a low risk of developing ischemic‐type biliary lesions. If validated, this observation might revolutionize DCD liver utilization. However, issues with bile collection, such as technical problems with bile duct cannulation, could lead to discarding usable livers. We agree with findings from the Cleveland group that the importance of bile production in the context of NMP is possibly overestimated.^22^

NMP provides the opportunity to explore multiple parameters, and it is still to be determined which can best predict posttransplant outcomes. We anticipate that future assessment methods will include more sophisticated techniques, including perfusate proteomic and metabolomic profiling to identify sensitive biomarkers, which could be used in conjunction with the proposed viability criteria to provide further objective measurement of liver functional integrity.^23^, ^24^, ^25^, ^26^ In this study, we also present the outcome of miRNA122 quantitation, frequently used as a marker of tissue injury. The assay system we developed was technically robust and well validated. We identified and used an appropriate control miRNA and included positive (spiked) controls. We were unable to show a difference in miRNA122 levels between the livers defined viable (LC) or nonviable (non‐LC). This suggests that although miRNA122 may correlate with the degree of tissue damage, it would not appear to be of value in the determination of liver function according to our proposed criteria.

Lactate is the intermediate metabolite of pyruvate within the glycolysis metabolic pathway. In NMP, hyperlactatemia is predominantly due to relative tissue hypoxia resulting from impaired liver blood flow and decreased gluconeogenesis. In this setting, lactate production may exceed its clearance and may be an indicator for real‐time liver function monitoring. Viability assessment based principally on lactate clearance offers several advantages compared with other proposed markers: lactate clearance can be measured 30‐90 minutes earlier than bile production, providing a particular advantage when using machines designed for relatively short perfusions; lactate can be measured sequentially, providing a trend, and the rate of decline in lactate concentration adjusted for mass of liver tissue (lactate/g) may be an even better parameter for characterizing the metabolic capacity of the liver compared with simple cutoff levels. This aspect is under active investigation by our group.

The comprehensive histopathological assessment reflected differences between the livers that were consistent with the grouping based on lactate clearance. The development of subtle zone 3 changes to hepatocytes/hepatocyte plates with loss of cell adhesion between them and loss of contact with the sinusoidal lining, features reminiscent of autolytic changes seen at postmortem, suggest that this is an ischemic injury modified by lack of tissue response. Hepatocyte glycogen stained by PAS was maintained at higher levels or increased in the LC compared with the non‐LC livers over the course of the perfusion, suggesting increased glycogen replenishment. Small‐droplet microvesicular steatosis has been seen to develop during cold storage and subsequently following reperfusion, suggesting that this may also be a response to ischemia/reperfusion injury.^16^ Taken together, these results support our hypothesis and suggest that grouping these discarded livers into viable and nonviable groups according to objective functional parameters has merit.

In implementing this novel strategy into our organ selection pathway, patient safety was the highest priority. We set an initial target of meeting criteria within 2 hours of starting NMP. We appreciate that some “nonviable” organs according to the proposed criteria may still be salvaged by delaying the cutoff for viability assessment or by increasing the required lactate value. Whether livers from a “gray zone” of organs achieving lactate levels of 2.5‐4 mmol/L later can be used, or if supplementary therapeutic interventions might allow safe transplantation of these organs is an important area of ongoing research.^27^

A limitation of our findings is incomplete perfusate transaminases and their correlation with the lactate measurements. Transaminase concentrations have often been used as a surrogate marker of hepatic injury related to the machine perfusion procedure and transplantation.^11^, ^28^ Because of progressive perfusate hemolysis during NMP, we were only able to obtain complete sets of perfusate transaminases from 4 perfusions (8, 10, 11, and 12). In each, there was a steady increase in ALT over the course of the 6‐hour perfusion (6851 IU in the liver that was successfully transplanted). Currently, we are unable to comment whether transaminase levels might be used as a reliable indicator of liver function or if they represent a snapshot of the extent of cellular injury that occurred prior to commencing NMP. Another limitation of the proposed criteria is that the primary focus is on function during the early posttransplant period, aiming to prevent early allograft dysfunction and primary nonfunction, but they do not provide any information concerning the likely longterm posttransplant outcome. We were unable to provide robust data regarding bile duct condition that could be compared in the context of the Groningen and Cambridge groups’ dedicated research on ischemic cholangiopathy.^29^, ^30^

The use of high‐risk organs remains globally low, with the principal reasons for rejecting livers being steatosis, poor organ flushing, and prolonged CITs. Although this proportion may differ between countries, these indications clearly imply clinicians’ fear of primary nonfunction. Ischemic cholangiopathy as a rationale for liver discard would be pertinent only to DCD donors, particularly for those with prolonged WITs. For DBD livers, however, the risk of developing cholangiopathy is insignificantly low.^9^ We believe the proposed criteria, focused primarily on the risk of primay nonfunction, might globally increase the utilization of currently wasted livers.

The proposed criteria are, to our knowledge, the first to be used successfully to select and transplant viable livers from the current pool of unused organs.^12^, ^31^ Having used these for over 3 years, we gained experience and developed confidence in the viability criteria, allowing us to progress to transplanting a subset of these originally discarded livers successfully.

These criteria were tested in a clinical pilot published previously, and all patients included in that series are well, with normal liver function and to date 3 years or longer of follow‐up. With our increased experience, we now believe the proposed criteria, used as a starting point for our subsequent work, including the Viability testing and transplantation of marginal livers (VITTAL) study, are conservative and can be further refined.^32^

In summary, this study introduces a composite of viability criteria including lactate concentration, bile production, and vascular flow patterns. The introduction of objective, real‐time methods of assessment are urgently required to address the underutilization of high‐risk livers. NMP may lead to considerable expansion of the donor pool available for transplantation. Although an assessment of viability is important to prevent early posttransplant graft failure, the effects on longterm transplant outcomes are yet to be determined.
